# Fire Source Range Localization Based on the Dynamic Optimization Method for Large-Space Buildings

**DOI:** 10.3390/s18061954

**Published:** 2018-06-15

**Authors:** Guoyong Wang, Xiaoliang Feng, Zhenzhong Zhang

**Affiliations:** 1 Luoyang Institute of Science and Technology, Luoyang 471000, China; standardwgy@163.com; 2 College of Electrical Engineering, Henan University of Technology, Zhengzhou 450001, China; 3 Bureau of Geophysical Prospecting, China National Petroleum Corporation, Zhuozhou 072751, China; zhangzhenzhong@cnpc.com.cn

**Keywords:** fire source localization, dynamic optimization, global information, the Range-Point-Range frame, the Range-Range-Range frame, sensor array

## Abstract

This paper is concerned to the fire localization problem for large-space buildings. Two kinds of circular fire source arrangement localization methods are proposed on the basis of the dynamic optimization technology. In the Range-Point-Range frame, a dynamic optimization localization is proposed to globally estimate the circle center of the circular arrangement to be determined based on all the point estimates of the fire source. In the Range-Range-Range frame, a dynamic optimization localization method is developed by solving a non-convex optimization problem. In this way, the circle center and the radius are obtained simultaneously. Additionally, the dynamic angle bisector method is evaluated. Finally, a simulation with three simulation scenes is provided to illustrate the effectiveness and availability of the proposed methods.

## 1. Introduction

In recent decades, more and more large-space buildings have been utilized as product storage and manufacture places, which are usually designed with complicated structures and are crowded with many kinds of materials and products. For these places, fire safety has become one of the most important and difficult problems, and increasing attention has been by governments, factories, firemen, researchers, and engineers [1,2,3,4,5,6]. For fighting against fire disasters, a critical important issue is how to determine the location of a fire source, in order to provide accurate information and necessary guidance for firefighting. Therefore, it is necessary to develop fire location methods to determine the fire source location as quickly and accurately as possible, for intelligent firefighting, especially in large-space buildings.

In fact, the fire source location method is one kind of wireless indoor positioning technique [7,8,9,10,11]. The existing fire source location methods are mainly developed on the basis of image processing technology, fiber-optic temperature measurement, temperature sensor arrays, and smoke sensor arrays. The fire location systems based on image information are commonly used for the large open spaces, and are easy impacted by barriers [12,13,14]. The fiber-based temperature measurement methods utilize the impacts of temperature on the anti-Stokes spectral lines in the Raman scattering process in an optical fiber to determine the fire sources position, which is suitable for tunnels and other such scenes, however, the cost of installation and maintenance of optical fiber is too expensive [15,16,17,18]. Moreover, several temperature field-based positioning methods aiming at environments, such as mines and forests, are given in [19,20,21,22]. These methods usually require that the fire releases more heat. Recently, the fire monitoring and location methods based on temperature/smoke sensor arrays have been paid increasing attention, in which the measurements are sometimes non-line-of-sight propagated. The fire source burning process produces the hot gases or smoke, which rise up to the ceiling and spread in a nearly circular shape [23,24,25,26,27,28]. With two temperature sensor arrays, a prototype system for determining the fire location was proposed in [18], in which three temperature sensors of a four-sensor array were utilized to estimate the angle of the fire source to the sensor array, and the fire source location can be obtained with the help of two estimated angles and the distance between two sensor arrays. This method was identified as the far-field mode in [24], due to the assumption that the distance between the fire source and the sensor array is much large than the distance intervals in the sensor array. In [24], a far-field fire location approach and a near-field method were presented and compared. In the near-field fire location method, the distance and the angle of the fire source to the sensor array were estimated simultaneously, with signal source localization of the planar wave fronts, but not the planar wave fronts, which was utilized in the far-field approach. The position estimation of the smolder source was realized in [28], using the smoke sensor arrays arranged in the planar circular mode. In [25], a fire location position algorithm was also developed with the signal source localization of a bilinear sensor array. As shown in [27,29], the output results of the above fire source location methods were a number of dispersedly estimated point coordinates of the fire source. The fire source point coordinate estimates were solved locally and independently with the sensor data sampled in the current time, but not the global sensor data sampled in the time series. Therefore, the clustering algorithm was introduced in the fire source localization method in the Range-Point-Range (RPR) frame in [27]. Additionally, two effective fire range estimation approaches were proposed in the Range-Range-Range (RRR) frame in [29]. The circle range estimates were obtained globally in the form of the inscribed circle and the circum-circle of a quadrangle, which was solved recursively using a dynamic angular bisector method. However, it did not discuss the optimality of the circle ranges solved by the dynamic angular bisector method in [29].

Inspired by the above discussion, in this paper, the method of determining the circle range which covers the fire source is developed based on the dynamic optimization theory. Firstly, in the RPR frame, all the point estimates of the fire source obtained by the fire source point location method are considering to obtain a circle center of the circular fire source range to be determined, the sum distance from which each fire point estimate is minimizes. The radius is the standard deviation of the distances from the circle center to every fire point estimate. Actually, compared with to the clustering algorithm in [27], this method determines the circle center and radius based on all the fire point estimates. Therefore, the circular fire source range is determined globally, according to [29]. However, the dynamic optimization problem solved in this algorithm is not recursive. When several new fire point estimates are obtained by using the current measurements of sensor arrays, all the obtained fire point estimates in the time series should be considered to determine the circle center and radius of the circular fire source range to be determined. Although the location accuracy can be improved along with the fire source range determining process, the computing complexity is increasing poor. In order to reduce the computing complexity of the dynamic optimization problem, a circular fire source range location method is also developed in the RRR frame. In [29], the quadrangle is solved by using the dynamic angle bisector method with global information, which, in this paper, is utilized to determine a new circum-circle, the circle center and radius of which is solved by minimizing the distances of the circle center to the four vertices of the given quadrangle. An interesting discovery is that the circle center is similar to the one obtained by the dynamic angle bisector method. It seems that the circle center obtained by using the dynamic angle bisector method is optimal in the sense of minimizing the distances of the circle center to the four vertices of the quadrangle solved with the global information.

The major contributions of this paper are three-fold. First, with all the fire point estimates, a circular fire source range estimation method is proposed in the RPR frame. As shown in [27], the clustering algorithms used to obtain the circular fire source range inevitably discard some fire point estimates. However, in this paper, the circular fire source range is solved globally by using a dynamic optimization algorithm based on all the point estimates of the fire source. Secondly, the above circular fire source range location method is developed in the RRR frame to reduce the computational complexity. The global angle information in time series are recursively utilized to obtain a quadrangle by using the dynamic angle bisector method. What is different with the method in [29] is that the circle center and the radius are determined by solving a non-convex optimization problem. In fact, the non-convex optimal problem can be treated as a standard to evaluate the dynamic angle bisector method. Additionally, the optimality of the dynamic angle bisector method is analyzed by the numeric examples in the simulation section.

It should be noted that, for the case where the angle information are not uniform on the two sides of the bisectors, the proposed circular fire source arrangement methods have better location results than the dynamic angle bisector method in [29], as illustrated in the simulation.

The rest of this paper is organized as follows: In Section 2, the fire location problem is presented, and a fire source point localization principle is briefly introduced. On this basis, two fire source range localization methods based on the dynamic optimization algorithm are proposed in Section 3. The first one is designed with the help of the fire point estimates in the RPR frame in Section 3.1, and in Section 3.2, the second method is developed with the quadrangle solved by the dynamic angle bisector method, which is studied in the RRR frame. In Section 4, a simulation with three different simulation scenes are provided to prove the effectiveness of the proposed methods. Finally, the conclusion of this paper is given in Section 5.

## 2. Problem Formulation

**Figure 1:**
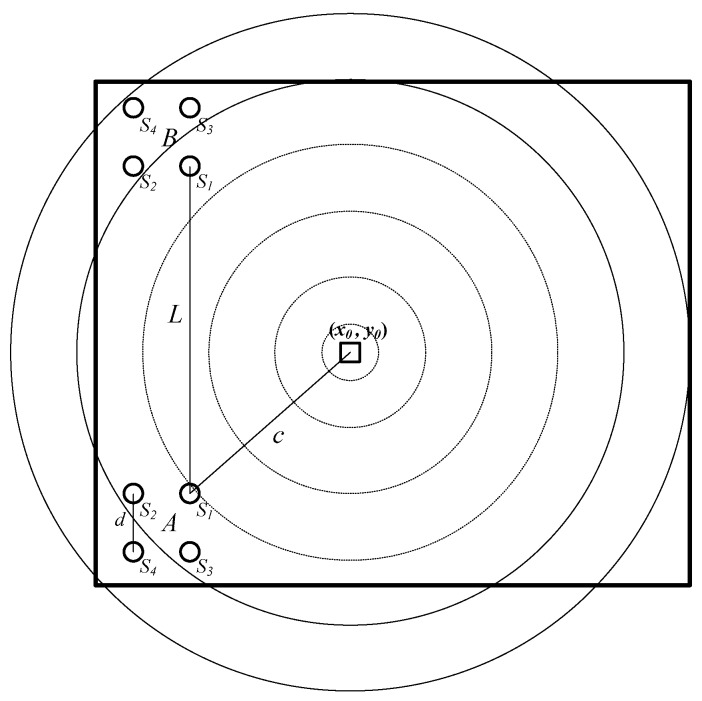
The fire source localization scene.

The structured fire scene studied in this paper is similar to the ones in [6,12,29]. An ignition fire source is considered in a large-space building with static wind. The fire source burning process produces the hot gases, which rise up to the ceiling and spread in a nearly circular shape at a constant current velocity from, and around, the center and form a hot upper layer [6,12,29], as shown in Figure 1. In Figure 1, two temperature sensor arrays are placed in different locations on the ceiling to monitor the air temperature. In each sensor array, four homogeneous temperature sensors are arranged in a square shape with the side length d. The distance between the two sensor arrays is L. The distances from the fire source to the two sensor arrays are $D_{1}$ and $D_{2}$, respectively. Then the expectation of the temperature field around the fire source can be described as [12]:

(1) $${E\{T(x,y,t)\}|}_{{\left(x-x_{0}\right)}^{2}+{\left(y-y_{0}\right)}^{2}=c^{2}}=f(t)$$

where $\left(x_{0},y_{0}\right)$ is the point coordinate of fire source and f(t) is the temperature function at location (x,y), the distance from which to the fire source is c.

For the structured fire scene described above, several effective fire source point location methods are proposed in [6]. Taking the famous far-field algorithm as an example, the main steps of the fire source point location scheme can be sketched as follows:

(1) *Delay Estimation*. As shown in Figure 1, there are different distances from the fire source to different temperature sensors. According to the temperature field expectation in Equation (1), the delay time of the same temperature time series spread to different sensors can be estimated by the correlation function method [12], or the gray relation analysis method [29].
(2) *Angle Estimation*. Denote $\tau_{i,j},\;(i,j=1,2,3,4)$ as the delay time from $S_{i}$ to $S_{j}$, $f_{A}$ as the sample rate of the temperature sensors, α(k) is an intersection angle crossed by the horizontal line and the line from the sensor array to the fire source, as shown in Figure 1. Based on the planar waves assumption of the far-field algorithm, for the sensor set $\left\{S_{1},S_{2},S_{3}\right\}$, one can obtain:
  (2) $$\left|v\right|\frac{\tau_{12}}{f_{A}}=d\cos(\alpha (k))$$
  (3) $$\left|v\right|\frac{\tau_{13}}{f_{A}}=d\sin(\alpha (k))$$
  then:
  (4) $$\hat{\alpha }(k)=\arctan\left(\frac{\tau_{12}}{\tau_{13}}\right),\;\hat{v}=f_{A}\frac{d\cos(\hat{\alpha }(k))}{\tau_{12}}$$

Similarly, for different sensor sets in the same sensor array, such as $\left\{S_{1},S_{2},S_{4}\right\}$ and $\left\{S_{1},S_{3},S_{4}\right\}$, different estimates of α(k) can also be obtained. In the sensor array, the sensor at $S_{1}$ is the reference node. For the sake of convenience, the angle estimate is denoted as ${\hat{\alpha }}_{i,j}(k)$, in which k indicates the sampled times, i∈{A,B} represents the different sensor arrays, and j=1,2,3 signifies the different sensor sets.

(3) *Fire Source Point Estimation*. For every combination $\left\{{\hat{\alpha }}_{A,j}(k),{\hat{\alpha }}_{B,l}(k)\right\},\;(j,l=1,2,3)$, the fire source point can be estimated as follows [12]:
  (5) $${\hat{x}}_{0,j,l}(k)=L\frac{\tan({\hat{\alpha }}_{B,l}(k))}{\tan({\hat{\alpha }}_{A,j}(k))+\tan({\hat{\alpha }}_{B,l}(k))}+x_{1}$$
  (6) $${\hat{y}}_{0,j,l}(k)=L\frac{\tan({\hat{\alpha }}_{B,l}(k))}{\tan({\hat{\alpha }}_{A,j}(k))+\tan({\hat{\alpha }}_{B,l}(k))}+y_{1}$$
  where $\left(x_{1},y_{1}\right)$ is the coordinate of $S_{1}$ in the sensor array A.

According to the fire source point estimation method given in Equations (5) and (6), nine estimates of the fire source can be solved at one time. Afterward, one can obtain a great deal of discretely ruleless estimate points. This kind of result limits the ability to guide firefighters. Therefore, some modified fire location methods are developed based on the fusion method [12,29]: the clustering algorithms [27]. In the next section, two kinds of location methods are designed to determine more accurate and compact ranges of the fire source, based on the above fire source point estimation method.

## 3. Main Results

### 3.1. Dynamic Optimization Localization Method in the RPR Frame

According to the fire source point estimation method mentioned in the above section, several ruleless discrete location points can be solved at one time. In order to fuse this location information into one estimate point, the mean of the three angle estimates for each sensor array is taken as the final angle estimate at one time, then the fusion fire source points are solved by Equations (5) and (6) in [12]. As noted in [29], this fusion result is solved only based on the sensor data at the current time, but not all of the sensor data in the time series. Therefore, in [27], a fire source range location method in the RPR frame is proposed based on an improved clustering algorithm. The fire source point estimates in the time series are utilized to determine a circular range which contains the real fire source with a large probability. However, this fire source circle range does not contain all the fire source point estimates in the clustering algorithm [29].

In this section, a fire source range location method is studied in the RPR frame, based on the dynamic optimization algorithm. All the estimates $\left({\hat{x}}_{0,j,l}(t),{\hat{y}}_{0,j,l}(t)\right)$, j,l=1,2,3; t=1,2,⋯,k obtained by Equations (5) and (6) contain some information of the fire source. Thus, all of them should be considered to deal with the globally determined circular range. Therefore, in this section, the circle center of the circular sharp arrangement to be determined is considered as the solution of the following optimization problem:

(7) $$\min\;sum\left({\left({\hat{x}}_{0}^{k}-x_{0}\right)}^{2}+{\left({\hat{y}}_{0}^{k}-y_{0}\right)}^{2}\right)$$

where $\left({\hat{x}}_{0}^{k},{\hat{y}}_{0}^{k}\right)$ is the matrix including all the fire source point estimates in the time series obtained by the fire source point estimation method mentioned in the above section.

It is indicated in Equation (7) that the sum of the distances between the circle center to be solved and every fire source point estimates in the time series should be the minimum.

Denote the solution of the optimization problem of Equation (1) as $\left({\hat{x}}_{0}(k),{\hat{y}}_{0}(k)\right)$. Take it as the circle center of the circular range to be determined, and take the standard deviation of the distances from $\left({\hat{x}}_{0}(k),{\hat{y}}_{0}(k)\right)$ to every fire source point estimate $\left({\hat{x}}_{0,j,l}(t),{\hat{y}}_{0,j,l}(t)\right)$, j,l=1,2,3; t=1,2,⋯,k as the radius of the circular range to be determined. Namely:

(8) $$r(k)=std\left\{{\left({\hat{x}}_{0,j,l}(t)-{\hat{x}}_{0}(k)\right)}^{2}+{\left({\hat{y}}_{0,j,l}(t)-{\hat{y}}_{0}(k)\right)}^{2}\right\},\;j,l=1,2,3;\;t=1,2,\cdots ,k$$

where, $\left({\hat{x}}_{0,j,l}(t),{\hat{y}}_{0,j,l}(t)\right),j,l=1,2,3;\;t=1,2,\cdots ,k$ are just the constituent elements of the matrix $\left({\hat{x}}_{0}^{k},{\hat{y}}_{0}^{k}\right)$.

In this way, a circular fire source range is determined by solving the optimization problem in Equation (7).

**Remark** **1.** *All the fire source point estimates in the time series are utilized to solve the dynamic optimization problem in Equation (7). Therefore, the circle center is solved based on the global information.*

However, the dynamic optimization problem solved in this algorithm is not recursive. When several new fire point estimates are obtained by using the current measurements of sensor arrays, all the obtained fire point estimates in the time series should be considered to determine the circle center and radius of the circular fire source range to be determined. Although the location accuracy can be improved along with the fire source range determining process, the computing complexity is increasing poor. In order to reduce the computing complexity of the dynamic optimization problem in the RPR frame, a circular fire source range location method is also developed in the RRR frame in the next subsection.

### 3.2. Dynamic Optimization Localization Method in the RRR Frame

In the fire source localization scene shown in Figure 1, for each sensor array i, i∈{A,B}, three angle estimates can be obtained at each time k, according to Equations (2) and (3). In the RRR frame [29], the up and down bounds of the angle estimate range can be obtained by using the statistical mean and variance of the three angle estimates:

(9) $${\hat{\alpha }}_{i}(k)=\frac{1}{3}\sum_{j=1}^{3}{\hat{\alpha }}_{i,j}(k),\;\delta_{i}^{2}=\frac{1}{2}\sum_{j=1}^{3}{\left({\hat{\alpha }}_{i,j}(k)-{\hat{\alpha }}_{i}(k)\right)}^{2}$$

(10) $${\hat{\alpha }}_{i}^{H}(k)={\hat{\alpha }}_{i}^{}(k)+\delta_{i}^{},\;{\hat{\alpha }}_{i}^{L}(k)={\hat{\alpha }}_{i}^{}(k)-\delta_{i}^{}$$

For the global angle estimates in the time series, the corresponding statistical mean and variance can be recursively calculated by:

(11) $${\hat{\alpha }}_{i}^{k}=\frac{{\hat{\alpha }}_{i}(k)+(k-1){\hat{\alpha }}_{i}^{k-1}}{k},\;{\left(\delta_{i}^{k}\right)}^{2}=\frac{{\left({\hat{\alpha }}_{i}(k)-{\hat{\alpha }}_{i}^{k}\right)}^{2}+(k-1){\left(\delta_{i}^{k-1}\right)}^{2}}{k-1}$$

Then, the up and down bounds of global angle estimate range can be given by:

(12) $${\hat{\alpha }}_{i}^{k,H}(k)={\hat{\alpha }}_{i}^{k}(k)+\delta_{i}^{k},\;{\hat{\alpha }}_{i}^{k,L}(k)={\hat{\alpha }}_{i}^{k}(k)-\delta_{i}^{k}$$

**Figure 2:**
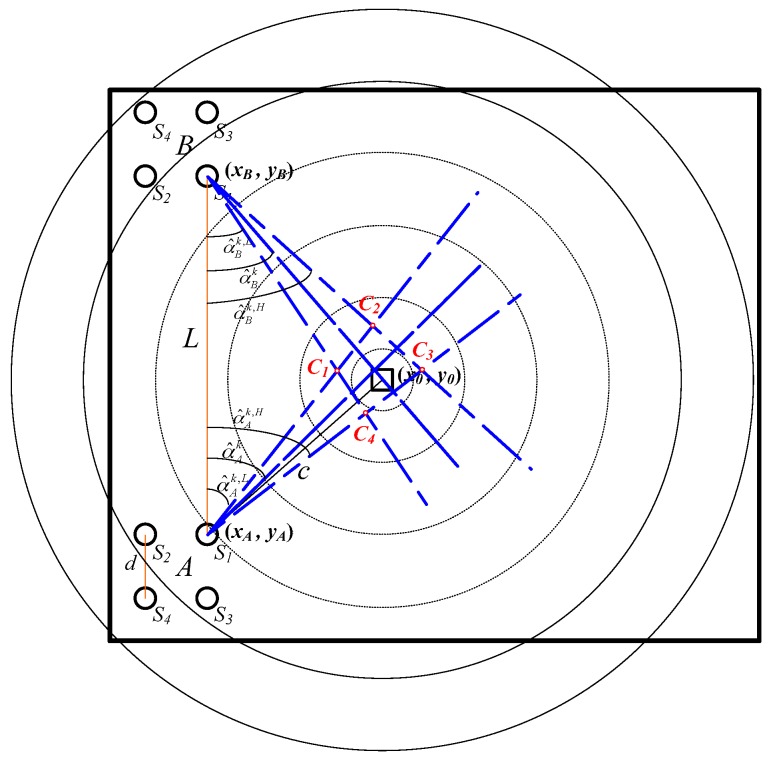
The fire source arrangement localization principle in the RRR frame.

The overlapped area of the global angle estimate ranges of the two sensor arrays contains the fire source with large probability, as shown in Figure 2. In [29], a dynamic angle bisector method was proposed to determine a circular fire source arrange, the circle center and radius of which were calculated on the basis of this overlapped area, which is a quadrangle. Actually, using the statistical features of the global data to obtain the quadrangle which contains the fire source with large probability is an excellent data compression method, which can also be used to reduce the computing complexity of the dynamic optimization problem in the proposed fire source range location method in Section 3.1.

As show in Figure 2, set the reference vertices’ coordinates of two sensor arrays as $\left(x_{A},y_{A}\right),\left(x_{B},y_{B}\right)$, then the four vertices $C_{j}(c_{j}^{k},d_{j}^{k}),\;(j=1,2,3,4)$ of the quadrangle overlapped by the two global angle estimate ranges can be solved by [29]:

(13) $$\{\begin{array}{l} c_{1}^{k}=\frac{\tan({\hat{\alpha }}_{A}^{k,L})x_{1}-\tan({\hat{\alpha }}_{B}^{k,H})x_{2}-y_{1}+y_{2}}{\tan({\hat{\alpha }}_{A}^{k,L})-\tan({\hat{\alpha }}_{B}^{k,H})};\;d_{1}^{k}=\frac{y_{2}/\tan({\hat{\alpha }}_{B}^{k,H})-y_{1}/\tan({\hat{\alpha }}_{A}^{k,L})-x_{2}+x_{1}}{1/\tan({\hat{\alpha }}_{B}^{k,H})-1/\tan({\hat{\alpha }}_{A}^{k,L})}\\ c_{2}^{k}=\frac{\tan({\hat{\alpha }}_{A}^{k,H})x_{1}-\tan({\hat{\alpha }}_{B}^{k,H})x_{2}-y_{1}+y_{2}}{\tan({\hat{\alpha }}_{A}^{k,H})-\tan({\hat{\alpha }}_{B}^{k,H})};\;d_{2}^{k}=\frac{y_{2}/\tan({\hat{\alpha }}_{B}^{k,H})-y_{1}/\tan({\hat{\alpha }}_{A}^{k,H})-x_{2}+x_{1}}{1/\tan({\hat{\alpha }}_{B}^{k,H})-1/\tan({\hat{\alpha }}_{A}^{k,H})}\\ c_{3}^{k}=\frac{\tan({\hat{\alpha }}_{A}^{k,L})x_{1}-\tan({\hat{\alpha }}_{B}^{k,L})x_{2}-y_{1}+y_{2}}{\tan({\hat{\alpha }}_{A}^{k,L})-\tan({\hat{\alpha }}_{B}^{k,L})};\;d_{3}^{k}=\frac{y_{2}/\tan({\hat{\alpha }}_{B}^{k,L})-y_{1}/\tan({\hat{\alpha }}_{A}^{k,L})-x_{2}+x_{1}}{1/\tan({\hat{\alpha }}_{B}^{k,L})-1/\tan({\hat{\alpha }}_{A}^{k,L})}\\ c_{4}^{k}=\frac{\tan({\hat{\alpha }}_{A}^{k,H})x_{1}-\tan({\hat{\alpha }}_{B}^{k,L})x_{2}-y_{1}+y_{2}}{\tan({\hat{\alpha }}_{A}^{k,H})-\tan({\hat{\alpha }}_{B}^{k,L})};\;d_{4}^{k}=\frac{y_{2}/\tan({\hat{\alpha }}_{B}^{k,L})-y_{1}/\tan({\hat{\alpha }}_{A}^{k,H})-x_{2}+x_{1}}{1/\tan({\hat{\alpha }}_{B}^{k,H})-1/\tan({\hat{\alpha }}_{A}^{k,L})} \end{array}$$

On this basis, the fire localization problem can be formulated to the following dynamic optimization problem, in which the circle center and radius of the circular fire region to be determined can be solved simultaneously:

(14) $$\begin{array}{l} \min\;r^{2}\\ s.t.\;{\left(x_{0}^{}-c_{j}^{k}\right)}^{2}+{\left(y_{0}^{}-d_{j}^{k}\right)}^{2}\leq r^{2}\;(j=1,2,3,4) \end{array}$$

where $\left(x_{0},y_{0}\right)$ and r are the center’s coordinate to be solved, respectively.

It should be noted that Equation (14) is only one of many optimization formulations and not the one and only. It is noted that Equation (14) is a non-convex optimization problem and it is difficult to solve. Thereby, in order to successfully solve this non-convex optimization, there is an available method to transform it to a set of convex optimization problems. In fact, the non-convex optimization problem given by Equation (14) can be expressed equivalently as the following set of four convex optimization problems, namely:

(15) $$\begin{array}{l} \min\;{\left(x_{0}^{}-c_{1}^{k}\right)}^{2}+{\left(y_{0}^{}-d_{1}^{k}\right)}^{2}\\ s.t.\;{\left(x_{0}^{}-c_{j}^{k}\right)}^{2}+{\left(y_{0}^{}-d_{j}^{k}\right)}^{2}\leq {\left(x_{0}^{}-c_{1}^{k}\right)}^{2}+{\left(y_{0}^{}-d_{1}^{k}\right)}^{2}\;(j=2,3,4) \end{array}$$

(16) $$\begin{array}{l} \min\;{\left(x_{0}^{}-c_{2}^{k}\right)}^{2}+{\left(y_{0}^{}-d_{2}^{k}\right)}^{2}\\ s.t.\;{\left(x_{0}^{}-c_{j}^{k}\right)}^{2}+{\left(y_{0}^{}-d_{j}^{k}\right)}^{2}\leq {\left(x_{0}^{}-c_{2}^{k}\right)}^{2}+{\left(y_{0}^{}-d_{2}^{k}\right)}^{2}\;(j=1,3,4) \end{array}$$

(17) $$\begin{array}{l} \min\;{\left(x_{0}^{}-c_{3}^{k}\right)}^{2}+{\left(y_{0}^{}-d_{3}^{k}\right)}^{2}\\ s.t.\;{\left(x_{0}^{}-c_{j}^{k}\right)}^{2}+{\left(y_{0}^{}-d_{j}^{k}\right)}^{2}\leq {\left(x_{0}^{}-c_{3}^{k}\right)}^{2}+{\left(y_{0}^{}-d_{3}^{k}\right)}^{2}\;(j=1,2,4) \end{array}$$

(18) $$\begin{array}{l} \min\;{\left(x_{0}^{}-c_{4}^{k}\right)}^{2}+{\left(y_{0}^{}-d_{4}^{k}\right)}^{2}\\ s.t.\;{\left(x_{0}^{}-c_{j}^{k}\right)}^{2}+{\left(y_{0}^{}-d_{j}^{k}\right)}^{2}\leq {\left(x_{0}^{}-c_{4}^{k}\right)}^{2}+{\left(y_{0}^{}-d_{4}^{k}\right)}^{2}\;(j=1,2,3) \end{array}$$

Denote the optimal solutions of Equations (15)–(18) as $\left(x_{0,j}^{k},y_{0,j}^{k}),\;(j=1,2,3,4\right)$, and set:

(19) $$r_{0,j}^{k}=\sqrt{{\left(x_{0,j}^{k}-c_{1}^{k}\right)}^{2}+{\left(y_{0,j}^{k}-d_{1}^{k}\right)}^{2}},\;j=1,2,3,4$$

Then, the optimal global solution on the radius of the circle to be determined is:

$$r_{0}^{k}=\min\left\{r_{0,1}^{k},r_{0,2}^{k},r_{0,3}^{k},r_{0,4}^{k}\right\}=r_{0,p}^{k}$$

where p∈{1,2,3,4}. The corresponding estimate of center’s coordinate of the expected circle is:

(20) $${\hat{x}}_{0}^{k}=x_{0,p}^{k},\;{\hat{y}}_{0}^{k}=y_{0,p}^{k}$$

Therefore, in the RRR frame, a fire source localization arrangement covering the fire source is presented with the dynamic optimization theory and the data compression method based on the statistical features of the global data.

**Remark** **2.** *Although the set of four convex optimization problems shown in Equations (15)–(18) can equivalently transform the non-convex optimization problem of Equation (14), but it cannot be directly solved with the CVX software package, which requires that constraints must be in the most simplified form. Thus, the optimization problems in (15)–(18) should be equivalently expressed as:*

(21) $$\begin{array}{l} \min\;{\left(x_{0}^{}-c_{1}^{k}\right)}^{2}+{\left(y_{0}^{}-d_{1}^{k}\right)}^{2}\\ s.t.\;2x_{0}^{}(c_{1}^{k}-c_{j}^{k})+2y_{0}^{}{\left(d_{1}^{k}-d_{j}^{k}\right)}^{2}\leq {\left(c_{1}^{k}\right)}^{2}+{\left(d_{1}^{k}\right)}^{2}-{\left(c_{j}^{k}\right)}^{2}-{\left(d_{j}^{k}\right)}^{2}\;(j=2,3,4) \end{array}$$

(22) $$\begin{array}{l} \min\;{\left(x_{0}^{}-c_{2}^{k}\right)}^{2}+{\left(y_{0}^{}-d_{2}^{k}\right)}^{2}\\ s.t.\;2x_{0}^{}(c_{2}^{k}-c_{j}^{k})+2y_{0}^{}{\left(d_{2}^{k}-d_{j}^{k}\right)}^{2}\leq {\left(c_{2}^{k}\right)}^{2}+{\left(d_{2}^{k}\right)}^{2}-{\left(c_{j}^{k}\right)}^{2}-{\left(d_{j}^{k}\right)}^{2}\;(j=1,3,4) \end{array}$$

(23) $$\begin{array}{l} \min\;{\left(x_{0}^{}-c_{3}^{k}\right)}^{2}+{\left(y_{0}^{}-d_{3}^{k}\right)}^{2}\\ s.t.\;2x_{0}^{}(c_{3}^{k}-c_{j}^{k})+2y_{0}^{}{\left(d_{3}^{k}-d_{j}^{k}\right)}^{2}\leq {\left(c_{3}^{k}\right)}^{2}+{\left(d_{3}^{k}\right)}^{2}-{\left(c_{j}^{k}\right)}^{2}+{\left(d_{j}^{k}\right)}^{2}\;(j=1,2,4) \end{array}$$

(24) $$\begin{array}{l} \min\;{\left(x_{0}^{}-c_{4}^{k}\right)}^{2}+{\left(y_{0}^{}-d_{4}^{k}\right)}^{2}\\ s.t.\;2x_{0}^{}(c_{4}^{k}-c_{j}^{k})+2y_{0}^{}{\left(d_{4}^{k}-d_{j}^{k}\right)}^{2}\leq {\left(c_{4}^{k}\right)}^{2}+{\left(d_{4}^{k}\right)}^{2}-{\left(c_{j}^{k}\right)}^{2}+{\left(d_{j}^{k}\right)}^{2}\;(j=1,2,3) \end{array}$$

*which can be directly solved with the CVX software package.*

**Remark** **3.** *Theoretically speaking, the angle bisector method in [29] subjectively fixes the circle center and the radius of the circular fire source arrangement to be determined, while the dynamic optimization method looks more reasonable because it can obtain a dynamic optimal solution of the estimate of circle center and the radius which, in the meantime, is also global. Clearly, there is a question that the subjective circle center cannot theoretically be guaranteed to be reasonable and perfect. The optimization problem in this section can be taken as a standard to evaluate the effectiveness of the angle bisector method. It should be noted that the circle center and the radius are estimated, respectively, although they have a very close connection. In fact, the circle center can be considered as a solution of the optimization problem that the minimum distance from a point to be determined in the quadrangle to cover all the four vertices, and the circum-circle radius estimated by the angle bisector method is the shortest one of the four distances from the vertices to the determined circle center, but not the distance solved by the last optimization problem. Therefore, there are some areas in the quadrangle that cannot be covered by the circum-circle estimated by the angle bisector method. For the dynamic optimal method in the RRR frame, the circle center and radius are estimated simultaneously in a consistent optimization standard. Thus, the circum-circle determined by the estimated circle center and radius cover the whole quadrangle. Therefore, the output of the dynamic optimization localization method in the RRR frame is another kind of circum-circle, which covers the whole quadrangle. It differs from the output of the angle bisector method with the circum-circle.*

**Remark** **4.** *Similar to the angle bisector method in [29], the dynamic optimal localization method in the RRR frame is sensitive to the statistical character of the measurement data on the fire position. For cases where the mean of the fire source point estimates are not zero, the location performances will be reduced to some extent. Nevertheless, the dynamic optimization localization method in the RPR frame has stronger robustness for the change of statistical characters of the measurement data.*

## 4. Simulation

In this section, a numerical simulation with three different simulation scenes are provided to prove the effectiveness and availability of the two proposed dynamic optimal localization methods. The simulation settings are similar to those set in [12,29], as shown in Table 1 and Table 2.

No matter the localization methods designed in the RPR frame or the RRR frame, the processes of delay time estimation and angle estimation are coincident, even for the principle of fire source point estimation. Therefore, the simulation of angle estimates do not influence the validity of the simulation results. The angle estimate simulation method in [29] is adopted to simplify the simulation process. Namely, several fire source point estimates are simulated firstly, and then the angle estimates can be computed according to the triangle principle.

In order to show the effectiveness and robustness of the proposed fire localization methods, they are compared with three other kinds of fire location methods in three different fire source localization simulation scenes.

**Figure 3:**
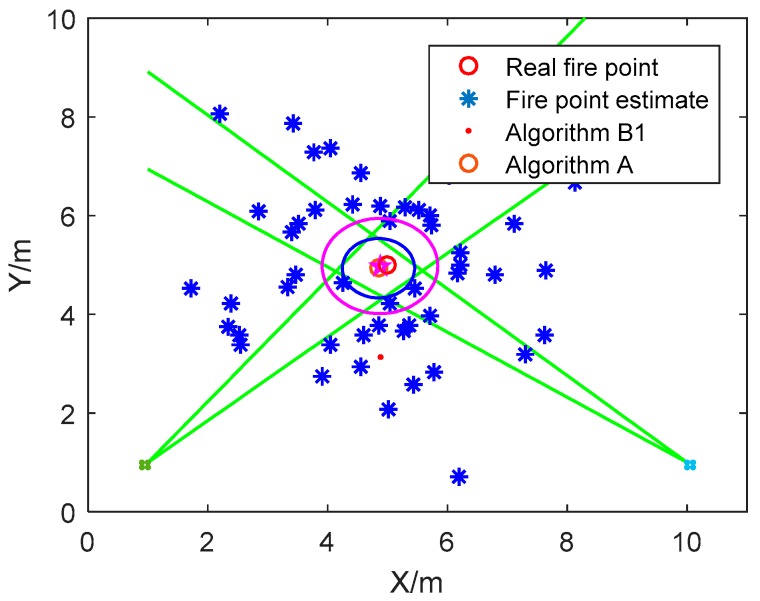
The circular fire source arrangement solved by Algorithm A and Algorithm B1.

**Simulation Scene 1**. The fire source point estimates are simulated based on the real fire coordinate by adding Gaussian noise. The estimation results of Algorithm A and Algorithm B1 are compared in Figure 3.

Although Algorithm A and Algorithm B1 can both be implemented with global data to obtain the circular fire source arrangement estimation, the estimated fire source arrangements are different. This is because the standard to guide the algorithm is different, which has been analyzed with detail in Remark 3. In addition, in Algorithm B1, it is subjective to take the standard deviation of the distances from the circle center to every fire source point estimate as the radius of the circular range to be determined. Thus, the circular fire source arrangement determined by Algorithm B1 is greater than Algorithm A, and the circle centers of the real fire point and its estimates by Algorithms A and B1 are shown in Table 3.

**Figure 4:**
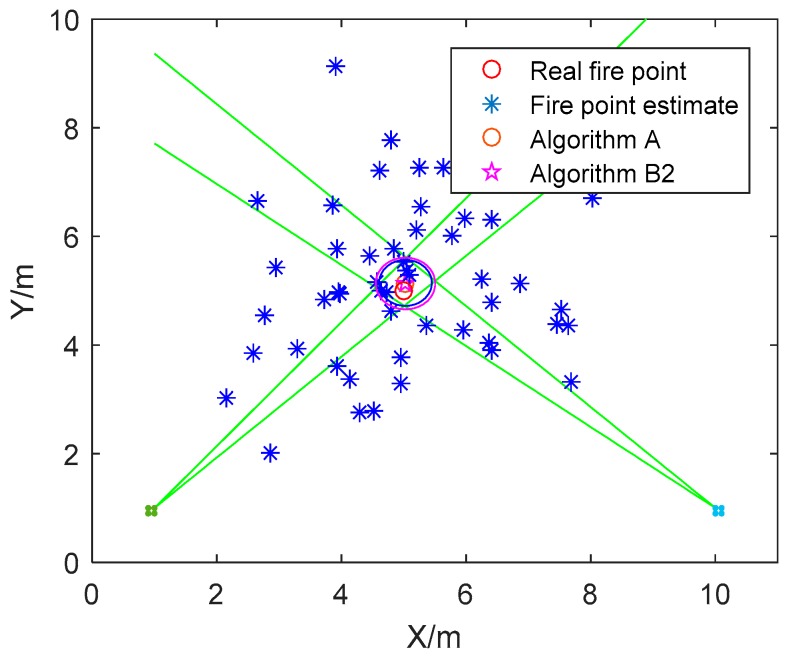
The circular fire source arrangement solved by Algorithm A and Algorithm B2.

**Simulation Scene 2.** The fire source point estimates are simulated based on the real fire coordinate by adding Gaussian noise. The estimation results of Algorithm A and Algorithm B2 are compared in Figure 4.

As illustrated in Figure 4, the circle centers of the circular fire source arrangement estimated by Algorithm A and Algorithm B2 are very similar, as shown in Table 4, the relative error is $O(10^{-2})$. Although the circles estimated by Algorithm A and Algorithm B2 are both the circum-circle of the quadrangle, the circum-circle obtained by Algorithm B2 can cover the whole quadrangle, while the one solved by Algorithm B2 cannot. The reason for this result has been analyzed in Remark 3.

**Simulation Scene 3**. The fire source point estimates are simulated based on the real fire coordinate by adding random mixed noise combined with Gaussian noise, Rayleigh noise, and sine wave signals. As shown in Remark 4, the dynamic optimal localization method in the RRR frame and the angle bisector method are sensitive to the statistical characters of the measurement data. Thus, the localization results are only compared by three robust localization methods: with the dynamic optimization localization method in the RPR frame, the localization method in the RPR frame with clustering technology, and the VB-ASCK method.

**Figure 5:**
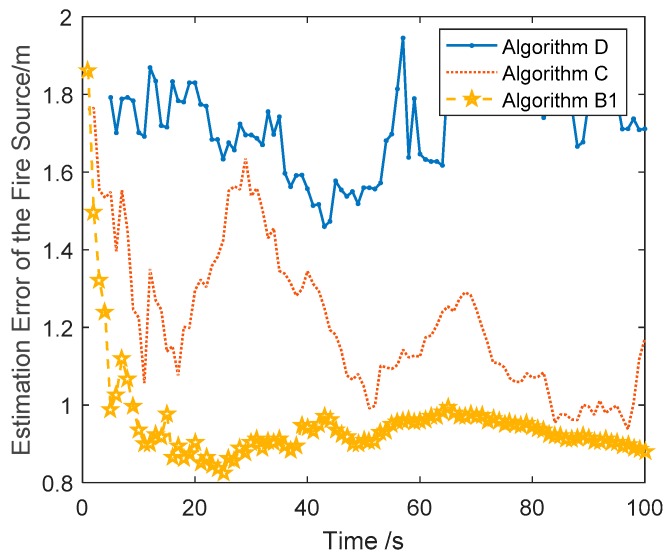
The estimation error curves of the fire source by the Algorithms B1, C, and D.

The simulation results given in Figure 5 are the average results of 100 Monte Carlo simulations. It is inevitable that the data from several measurements cannot be utilized to estimate the circle center and radius of the circular fire source arrangement. Therefore, the other two localization methods, with global measurement data, have better accuracy than Algorithm D. In Algorithm C, the mixed noise is taken as Gaussian noise with unknown covariance. Although the specific variational Bayesian technology is used to adaptively estimate the unknown covariance, the estimation accuracy of Algorithm C is worse than that of the dynamic optimization localization method in the RPR frame. As shown in Table 5, the mean estimation error of Algorithm D is 1.7, while that of Algorithm C is 1.2 and that of Algorithm B1 is 0.9.

## 5. Conclusions

In this paper, two circular fire source arrangement localization methods are proposed on the basis of the dynamic optimization technology. In the RPR frame, the dynamic optimization localization method is global, but not recursive, so the computing complexity of the dynamic optimization process is increasingly poor. Inspired by the data compression technology in the angle bisector method, a dynamic optimization localization method is developed by solving a non-convex optimization problem. The non-convex optimization problem can be treated as a standard to evaluate the dynamic angle bisector method. It should be noted that the circle radius is subjectively obtained by the dynamic optimization localization method. How to build an optimal index to solve the circle radius and the circle center optimally and synchronously is still an open topic for future research.

## Figures and Tables

**Table 1:** The simulation settings.

Simulation Background	Simulation Setting
The length of the buildings	11 m (meters)
The width of the buildings	11 m
the real point of fire source	(5 m, 5 m)
The distance between the two temperature sensor arrays	L=9 m
The distance between the two sensors in a array	$d_{0}=0.1\;m$
The coordinate of the reference node of sensor array A	(0.5 m, 1 m)
The coordinate of the reference node of sensor array B	(1 m, 10 m)
The sampling frequency	500 Hz

**Table 2:** The abbreviations of the algorithms used in this section.

The Algorithms	Abbreviations
The angle bisector method with the circum-circle	Algorithm A
The dynamic optimization localization method in the RPR frame	Algorithm B1
The dynamic optimization localization method in the RRR frame	Algorithm B2
The localization method based on VB-ASCKF in the RRR frame	Algorithm C
The localization method in the RRR frame with clustering technology	Algorithm D

**Table 3:** The circle centers of real fire point and its estimates by Algorithms A and B1.

The Real Fire Point	Algorithm A	Algorithm B1
(5, 5)	(4.849, 4.935)	(4.873, 4.98)

**Table 4:** The circle centers of real fire points and its estimates by Algorithms A and B2.

The Real Fire Point	Algorithm A	Algorithm B2
(5, 5)	(5.027, 5.144)	(5.027, 5.133)

**Table 5:** Mean estimation error of the fire source by the Algorithms B1, C, and D.

The Algorithms	Algorithm B1	Algorithm C	Algorithm D
Mean estimation error	0.9	1.2	1.7
